# The Prospective Indirect Effect of Mathematics Interest in the Relationship Between Mathematics Anxiety and Self-Concept: A Two-Wave Longitudinal Study

**DOI:** 10.3390/bs16050733

**Published:** 2026-05-09

**Authors:** Sedef Çelik Demirci, Ümit Kul, Samet Korkmaz

**Affiliations:** 1 Department of Mathematics and Science Education, Faculty of Education, Artvin Coruh University, 08100 Artvin, Türkiye; umitkul@artvin.edu.tr; 2 Department of Educational Sciences, Faculty of Humanities and Social Sciences, Karabuk University, 78050 Karabuk, Türkiye; sametkorkmaz@karabuk.edu.tr

**Keywords:** mathematics anxiety, mathematics interest, academic self-concept, two-wave cross-lagged indirect effect, middle school, Control-Value Theory

## Abstract

Although math anxiety (MA), math interest (MI), and math self-concept (SC) have been investigated separately in the literature, their simultaneous examination within a longitudinal framework remains limited. The present study investigated the longitudinal indirect effect of MI in the relationship between MA and SC, grounded in Control-Value Theory (CVT). A two-wave cross-lagged panel design was employed with a four-month measurement interval. The final analytic sample comprised 543 Turkish middle school students (Grades 6-8). Structural equation modeling (SEM) with 5000 bootstrap resamples was used to test the hypothesized indirect associations, with autoregressive controls included for all constructs. Measurement model fit was confirmed separately for each wave prior to structural estimation. Results indicated that MA at Time 1 negatively predicted both MI (β = −0.157, *p* < 0.01) and SC (β = −0.150, *p* < 0.01) at Time 2, while MI at Time 2 positively predicted SC at Time 2 (β = 0.626, *p* < 0.01). The indirect effect of MA on SC via MI was statistically significant (β = −0.098, 95% CI [−0.167, −0.034]), supporting a prospective indirect association. These findings demonstrate that MI functions as an active motivational mechanism through which early anxiety erodes students’ mathematical self-perceptions over time, and underscore the importance of interventions that simultaneously target MA reduction and interest development to protect SC in early adolescence.

## 1. Introduction

Math anxiety (MA), broadly defined as a feeling of tension and apprehension that interferes with the manipulation of numbers and the solving of mathematical problems (Richardson & Suinn, 1972), is among the most extensively studied affective constructs in educational psychology (Dowker et al., 2016). Meta-analytic evidence consistently documents its negative association with mathematics achievement across educational levels (Barroso et al., 2021; Caviola et al., 2022). Large-scale international assessments further indicate that approximately one in three students reports elevated levels of MA (OECD, 2013). In the Turkish context, Programme for International Student Assessment (PISA) results similarly reveal persistent patterns of underperformance and disengagement in mathematics, highlighting the need to understand the affective mechanisms underlying these trends (Kul et al., 2024). Beyond its direct effects on achievement, MA is associated with broader motivational consequences. Students experiencing high anxiety tend to progressively disengage from mathematical activities, restrict their perceived career options, and are substantially less likely to pursue STEM-related pathways (Ahmed, 2018; Cuder et al., 2024). Within Pekrun’s (2006) control–value theory (CVT), these outcomes emerge when anxiety, conceptualized as a negative activating emotion, undermines students’ appraisals of competence and the intrinsic value of mathematical activities. This process can initiate a self-reinforcing cycle characterized by avoidance, reduced engagement, and increasingly negative self-perceptions. Such dynamics are particularly pronounced during early adolescence, a developmental period marked by intensified social comparison, rising performance demands, and increasing domain differentiation of motivational beliefs (Eccles et al., 1993). Despite this theoretical framework, the psychological mechanisms through which MA undermines students’ academic self-concept over time remain incompletely understood, particularly in non-Western middle school contexts where longitudinal evidence is limited.

The present study integrates three complementary theoretical frameworks to explain this process. CVT posits that achievement emotions arise from students’ appraisals of control over outcomes and the subjective value of those outcomes. Anxiety, as a negative activating emotion, can disrupt control appraisals and interfere with the positive activity emotions, including interest, that sustain engagement. Expectancy-value theory (EVT; Eccles et al., 1983; Wigfield & Eccles, 2000) specifies that competence beliefs and subjective task values jointly determine motivation, effort, and persistence; when MA erodes competence perceptions, intrinsic task value, the motivational core of interest, declines correspondingly. Self-determination theory (SDT; Deci & Ryan, 2000) complements these accounts by proposing that sustained intrinsic motivation depends on the satisfaction of three basic psychological needs: autonomy, competence, and relatedness. Anxiety, by threatening perceived competence and autonomy, undermines the intrinsic motivation underlying interest and, in turn, students’ academic self-concept.

Academic self-concept in mathematics (SC), broadly defined as students’ domain-specific beliefs about their own mathematical competence (Marsh, 1986), represents a central determinant of sustained engagement, effort investment, and persistence in mathematics learning (Marsh & Martin, 2011; Wu et al., 2021). Longitudinal studies consistently show that MA negatively predicts subsequent academic self-concept, even after controlling for prior levels of self-concept (Ahmed et al., 2012; Gunderson et al., 2018; J. Zhang et al., 2023). A recent meta-analytic synthesis spanning childhood through adolescence confirmed that the anxiety–self-concept association is developmentally robust, although its magnitude varies across developmental stages and academic domains (Brumariu et al., 2023). These reciprocal dynamics may emerge early in formal schooling, particularly during periods of rapid development in competence beliefs, when maladaptive motivational patterns contribute to rising anxiety and declining self-perceptions, reinforcing negative cycles or shaping more adaptive trajectories depending on initial competence beliefs (Gunderson et al., 2018). Consistent with CVT, Forsblom et al. (2022) demonstrated in a three-wave longitudinal study that the effects of achievement on emotional experiences were fully mediated by perceived competence, highlighting the central role of self-concept appraisals within the anxiety–engagement cycle. Pinxten et al. (2014) extended this line of inquiry to mathematics specifically, demonstrating longitudinal reciprocal effects between enjoyment and perceived competence on mathematics effort and achievement, and highlighting that interest (enjoyment) and SC (competence) constitute empirically distinct but mutually reinforcing dimensions of mathematics motivation. Nevertheless, the motivational pathway through which anxiety initially erodes SC has not yet been examined within a simultaneous three-construct longitudinal framework.

Mathematics interest (MI), conceptualized as a positive affective orientation toward mathematics encompassing enjoyment, curiosity, and intrinsic motivation to engage with mathematical content (Hidi & Renninger, 2006), represents a theoretically plausible mediating mechanism in this process. Within CVT, anxiety is expected to suppress value appraisals and thereby diminish positive activity emotions such as enjoyment and interest. Supporting this mechanism, Forsblom et al. (2022) showed that perceived value prospectively predicted enjoyment across three annual measurement waves, whereas low perceived competence predicted boredom and anger. EVT similarly proposes that intrinsic task value, of which interest is a central component, operates alongside competence beliefs to determine effort and persistence. Empirical evidence further indicates that MI declines substantially during the middle school years (Sakaki et al., 2024). This decline often coincides with increases in MA, as longitudinal studies show that trajectories characterized by persistently high or increasing anxiety are associated with reduced interest and a lower likelihood of pursuing STEM careers (Ahmed, 2018; Cribbs et al., 2021). Conversely, interest may also play a formative role in shaping students’ academic self-concept. Broda et al. (2023) identified an at-risk motivational profile characterized by high anxiety, low interest, and low self-concept, which was associated with the poorest mathematics outcomes. Similarly, D. Zhang and Wang (2020) demonstrated that MI mediated the relationship between anxiety and mathematics achievement among Chinese middle school students. Nevertheless, Wu et al. (2021) noted that although academic interest is positively associated with both achievement and academic self-concept, the extent to which interest accounts for their relationship remains unclear. Collectively, these findings suggest a sequential process in which anxiety diminishes interest, and reduced interest subsequently undermines academic self-concept. However, this proposed pathway has not yet been examined as a unified longitudinal framework incorporating all three constructs simultaneously.

One important methodological reason for this gap is that these constructs demonstrate substantial autoregressive stability over time, which limits the residual variance available for detecting cross-lagged indirect effects. For instance, J. Zhang et al. (2023), in a three-wave cross-lagged study, documented SC stabilities of β = 0.93 and β = 0.71 across successive measurement intervals, and anxiety stabilities of β = 0.86 and β = 0.54, indicating that prior scores account for the majority of subsequent variance and leave limited opportunity for indirect effects to emerge. Consistent with this pattern, Gogol et al. (2016) showed that cross-construct developmental effects across two waves tend to be small and inconsistent, which helps explain why pairwise longitudinal designs frequently produce modest estimates of indirect associations. To date, most research has examined these constructs either in pairs (Gunderson et al., 2018; Wang et al., 2020) or within cross-sectional designs (Broda et al., 2023; Munoz et al., 2025). Consequently, the full anxiety–interest–self-concept sequence has not been tested simultaneously within a longitudinal framework. The present study addresses this limitation by employing a two-wave cross-lagged panel design with 543 Turkish middle school students in Grades 6 through 8. The four-month interval between measurements was selected to capture motivational change during early adolescence. Turkish middle school students represent a contextually relevant population given consistently elevated levels of MA and mathematics achievement below expected benchmarks reported in national assessments and scale-adaptation studies (Kul et al., 2024). Accordingly, this study provides one of the first longitudinal examinations of MI as an indirect mechanism linking MA to SC. Based on the theoretical and empirical evidence reviewed above, the following hypotheses were formulated:

**H1.**  *MA at T1 will negatively predict SC at T2, controlling for prior levels of SC.*

**H2.**  *MA at T1 will negatively predict MI at T2, controlling for prior levels of MI.*

**H3.**  *MI at T1 will positively predict SC at T2, controlling for prior levels of SC.*

**H4.**  *There will be a significant indirect effect of T1 MA on T2 SC through T2 MI, such that higher MA at T1 predicts lower MI at T2, which in turn relates to lower SC at T2.*

## 2. Literature Review

### 2.1. Math Anxiety

As formally introduced by Richardson and Suinn (1972), MA is defined as a state of tension that interferes with number manipulation and problem solving across a variety of ordinary and academic contexts. Subsequent research established the multidimensional nature of MA: a widely adopted two-factor model distinguishes learning mathematics anxiety, experienced during mathematical instruction, from mathematics evaluation anxiety, triggered during formal assessment (Carey et al., 2017). This distinction informed the measurement framework of the present study. MA is empirically distinguishable from general test anxiety, retaining domain-specific variance attributable to the unique cognitive and evaluative demands of mathematics (Ashcraft & Moore, 2009). Its consequences extend beyond impaired performance to include reduced working memory capacity during problem solving, avoidance of voluntary mathematical engagement, and restricted vocational aspirations (Ashcraft, 2002; Justicia-Galiano et al., 2017). A meta-analytic synthesis by Barroso et al. (2021) reported a weighted mean correlation of r = −0.28 between MA and achievement, confirming the stability and educational significance of this association. Chang and Beilock (2016) further demonstrated that the relationship between MA and performance is sensitive to motivational processes, particularly students’ engagement and interest in mathematics. Longitudinal evidence from the five-wave PALMA study (German adolescents, Grades 5–9) demonstrates that the relationship between anxiety and achievement is bidirectional: negative emotions, including anxiety, prospectively predicted lower grades and test scores, while lower achievement predicted higher subsequent anxiety (Pekrun et al., 2017). In cross-cultural contexts, similar structural distinctions have been observed. For example, Kul et al. (2024) confirmed the psychometric distinctiveness of learning and evaluation anxiety profiles in a Turkish early adolescent sample. Likewise, Cuder et al. (2024), in a three-year longitudinal study of Italian middle school students, found that Grade 7 anxiety emerged as the strongest unique predictor of STEM track avoidance, exceeding the predictive power of prior achievement and self-efficacy. Within motivational frameworks, particularly EVT, these consequences can be interpreted as reflecting the erosion of intrinsic task value and competence beliefs that are central to sustained mathematical motivation. Thus, MA functions not merely as an emotional state but as a persistent motivational threat that progressively undermines students’ willingness and capacity to engage with mathematics.

### 2.2. Academic Self-Concept in Mathematics

Academic self-concept refers to students’ domain-specific evaluative beliefs about their own academic ability (Marsh, 1986). In mathematics, it encompasses judgements of competence relative to peers and a broader identity as a mathematics learner. It is theoretically and empirically distinguishable from self-efficacy, which reflects task-specific confidence, whereas self-concept develops through cumulative social comparison and represents a more stable, identity-related perception (Arens et al., 2011; Bong & Skaalvik, 2003; Marsh & Martin, 2011). Reciprocal effects models demonstrate that academic self-concept and achievement mutually reinforce one another across time (Marsh & Martin, 2011; Wu et al., 2021). Gogol et al. (2016), using nested-factor longitudinal modeling, demonstrated that subject-specific self-concept is hierarchically structured with general and domain-specific components, exhibits substantial differential stability, and is structurally linked to interest and anxiety, positioning self-concept at the center of the motivational network examined in this study. Longitudinal studies consistently indicate that MA negatively predicts subsequent self-concept. For example, Ahmed et al. (2012) documented reciprocal negative relations between math self-concept and math anxiety across a single academic year, and J. Zhang et al. (2023) replicated the anxiety-to-self-concept pathway in a three-wave design. A recent meta-analysis by Brumariu et al. (2023) further confirmed the robustness of this association across developmental periods. Van der Beek et al. (2017) further demonstrated that self-concept fully mediated the relationship between mathematics achievement and achievement emotions. Gunderson et al. (2018) showed these anxiety–self-concept cycles are operative as early as Grades 1 and 2, underscoring that early competence experiences set in motion motivational trajectories that extend through middle school and beyond. Within EVT, SC represents the domain-specific competence belief that, together with intrinsic task value, serves as a proximal determinant of students’ achievement-related choices and persistence. Accordingly, SC is positioned as a central outcome in motivational models of mathematics learning.

### 2.3. Math Interest

Interest is conceptualized as both a situationally induced psychological state and a relatively stable individual predisposition toward particular content, developing through repeated, positively valenced engagement (Hidi & Renninger, 2006; Krapp, 2002). In mathematics, it reflects enjoyment, curiosity, and intrinsic motivation to engage with mathematical material. SDT provides a complementary account: when students’ basic psychological needs for autonomy, competence, and relatedness are satisfied, they engage with mathematical tasks volitionally and with genuine curiosity; conversely, when anxiety threatens perceived competence and autonomy, intrinsic motivation, and with it interest, erodes. This SDT perspective specifies the need-level mechanisms through which MA suppresses MI, linking the emotional account offered by CVT to the motivational structure described by EVT. Within CVT, interest is a product of positive control and value appraisals; anxiety, by eroding both, systematically suppresses interest and the positive activity emotions that sustain engagement (Pekrun, 2024).

Empirical evidence strongly supports these theoretical assertions. Forsblom et al. (2022) provided three-wave longitudinal evidence: perceived value prospectively predicted enjoyment across consecutive annual assessments, while low perceived competence predicted boredom and anger. MI follows a well-documented declining trajectory across middle school (Sakaki et al., 2024), linked to simultaneous increases in anxiety and declines in perceived competence. This decline has meaningful downstream consequences. For instance, D. Zhang and Wang (2020) showed that interest mediated the anxiety–achievement relationship in Chinese middle school students, and Denner et al. (2019), in a cross-lagged study, found that lower initial academic self-concept predicted greater subsequent declines in interest across the elementary-to-middle school transition. Broda et al. (2023) identified a high-risk motivational profile combining elevated anxiety, diminished interest, and low self-concept associated with the poorest mathematics outcomes. Furthermore, Munoz et al. (2025) demonstrated through structural equation modeling that MI mediates the anxiety–STEM-belonging pathway. Similarly, Cribbs et al. (2021) showed that mathematics identity fully mediated the relationship between anxiety and STEM career interest, confirming that interest-related constructs function as key motivational bridges in the anxiety–self-perception chain.

### 2.4. The Mediating Role of Math Interest

The theoretical and empirical evidence reviewed above supports a sequential motivational process in which MA erodes students’ intrinsic engagement with mathematics, and diminished MI in turn attenuates SC. Schukajlow et al. (2023), in a comprehensive cross-theoretical synthesis, argue that integrating CVT, EVT, and SDT provides a comprehensive account of how emotional and motivational processes jointly shape academic outcomes in mathematics. This conclusion is further supported by a systematic review and meta-analysis showing that achievement emotions represent among the most robust affective predictors of mathematics performance (Schoenherr et al., 2025). Despite this convergent theoretical support, the simultaneous examination of these prospective indirect associations within a three-construct longitudinal framework remains largely unexplored. Prior longitudinal studies have examined pairwise relationships in isolation, including anxiety predicting self-concept (J. Zhang et al., 2023), interest mediating anxiety and achievement (D. Zhang & Wang, 2020), and self-concept predicting interest decline (Denner et al., 2019). Studies including all three constructs have relied primarily on cross-sectional designs (Broda et al., 2023; Munoz et al., 2025). The high autoregressive stability of these constructs (Gogol et al., 2016; J. Zhang et al., 2023) further necessitates measurement intervals aligned with the pace of motivational change.

The present study addresses this gap by providing one of the first longitudinal examinations of MI as an indirect mechanism linking MA to SC, utilizing a two-wave cross-lagged panel design with a four-month interval among Turkish middle school students. Specifically, CVT contributes the emotional mechanism by explaining how negative activating emotions erode the control and value appraisals that sustain interest. EVT contributes the motivational structure by describing how diminished competence beliefs and task value jointly reduce persistence and engagement. SDT contributes the need-level foundation by explaining why interest depends on the satisfaction of basic psychological needs that anxiety systematically threatens. Taken together, these frameworks generate a convergent prediction: MA will prospectively suppress MI, and diminished MI will in turn attenuate SC.

## 3. Method

### 3.1. Participants and Procedure

This study employed a two-wave longitudinal panel design. Data were collected at two time points during the 2024 academic year: Time 1 (T1) at the beginning of the semester and Time 2 (T2) at the end of the same semester, with an interval of approximately four months between assessments. This interval was selected to be long enough to detect meaningful motivational change while remaining within a single academic year, thereby minimizing the influence of grade-level transitions or curriculum discontinuities. Questionnaires were administered in paper-and-pencil format by the researchers in classroom settings, and completed forms were collected immediately after administration. Before data collection, all necessary approvals were obtained from the relevant institutional and ethics committees. The study was conducted in accordance with the ethical standards of the 1964 Helsinki Declaration and its subsequent revisions. Participation was voluntary, and written informed consent was obtained from the parents of all participating middle school students; students also provided their own assent.

A total of 623 students participated at T1 and 555 at T2. To match data across the two time points, a coding system was applied in which students provided their student number, grade level, and classroom (e.g., 6/A, 7/B); this information was used solely for data linkage and not for identification. The final analytic sample comprised 543 middle school students (Grades 6–8) who participated at both time points and whose data could be matched reliably. Grade 6 was represented by 110 students, Grade 7 by 214 students, and Grade 8 by 219 students. Attrition between waves was primarily attributable to student absence on the day of the second assessment, school transfer, exam-period conflicts, scheduling changes that precluded administration in certain classrooms, and incomplete questionnaire responses.

### 3.2. Measures

#### 3.2.1. Mathematics Anxiety Scale

MA was assessed using the Modified-Abbreviated Math Anxiety Scale (MAMAS; Carey et al., 2017), adapted and validated for Turkish middle school students by Kul et al. (2024). The scale comprises nine items organized into two subscales: Mathematics Learning Anxiety (MLA; five items) and Mathematics Evaluation Anxiety (MEA; four items). Items are rated on a five-point Likert scale ranging from 1 (low anxiety) to 5 (high anxiety). Sample items include “I feel anxious when starting a new topic in mathematics.” In the Turkish adaptation, the overall scale demonstrated high internal consistency (α = 0.90). In the present study, subscale internal consistency coefficients were satisfactory across measurement waves. At Time 1, MLA demonstrated acceptable reliability (α = 0.76), whereas MEA showed adequate reliability (α = 0.77). At Time 2, MLA again demonstrated acceptable reliability (α = 0.76), and MEA showed good internal consistency (α = 0.80). These results indicate stable reliability across time points despite the relatively small number of items per subscale. Because mathematics anxiety was conceptualized as a multidimensional construct consisting of two theoretically grounded components, parcel-level subscale scores (MLA and MEA) were specified as indicator variables of the latent MA construct in the structural model. This approach is consistent with recommended practices in parcel-based confirmatory factor analysis when theoretically meaningful subdimensions are available (Little et al., 2002).

#### 3.2.2. Mathematics Interest Scale

Mathematics interest was measured using the Mathematics Interest Scale, comprising three items adapted from the OECD (2003), assessment framework, which has demonstrated adequate psychometric properties across diverse international middle school populations. The scale uses a four-point Likert format ranging from 1 (strongly disagree) to 4 (strongly agree). Items assess students’ affective engagement with and intrinsic orientation toward mathematics, including enjoyment and willingness to engage with mathematical activities (e.g., “I enjoy working with mathematics”). This wording is consistent with established large-scale assessments in which expressions such as enjoy and working with mathematics are interpreted by middle school students as engagement in typical mathematical tasks such as solving problems or completing exercises.

Internal consistency in the present sample was high (α = 0.93), indicating strong reliability despite the relatively small number of items. Prior validation studies using similar item sets with middle school populations have supported the unidimensional structure of MI, and CFA in the present study likewise supported this structure. Given the scale’s unidimensional structure, individual items were specified as observed indicators of a single latent MI construct within the measurement model. Measurement invariance across time points was tested to ensure that the MI construct was interpreted consistently across waves.

#### 3.2.3. Academic Self-Concept Scale

Following Marsh et al. (2019), the scale is conceptually and empirically distinguished from self-efficacy by its retrospective, descriptive, and socially comparative nature, justifying its separate assessment in the present study. SC was assessed using three items drawn from the OECD (2003) framework (e.g., “Mathematics is one of my best subjects”; “In my mathematics class, I understand even the most difficult work”). No subscales were present; all items loaded on a single factor. Items are rated on a five-point scale ranging from 1 (not at all true of me) to 5 (completely true of me) and reflect students’ self-perceptions of their mathematical competence and ability relative to their peers. The scale demonstrated high internal consistency (α = 0.87). Individual items served as indicator variables in the measurement model.

### 3.3. Data Analysis

Descriptive statistics and inter-variable correlations were computed using IBM SPSS v.19 Statistics. Internal consistency coefficients (Cronbach’s α) were computed in R (version 4.5.1). The longitudinal framework was tested using structural equation modeling (SEM) implemented in IBM AMOS. The analytical strategy proceeded in two stages. In the first stage, the measurement model for each wave was evaluated separately through CFA to establish construct validity and acceptable fit prior to structural modeling. In the second stage, a cross-lagged panel model was estimated to examine the prospective indirect associative role of MI in the MA–SC relationship.

In the structural model, MA (T1) was specified as the independent variable (X), MI (T2) as the intervening mechanism (M), and SC (T2) as the outcome variable (Y). T1 scores for each construct were included as autoregressive controls to account for baseline levels and stability. Specifically, the model examined (a) the cross-lagged path from T1 MA to T2 MI, controlling for T1 MI; (b) the concurrent path from T2 MI to T2 SC, controlling for T1 SC and T1 MI; and (c) the direct cross-lagged path from T1 MA to T2 SC, controlling for T1 SC. This specification is consistent with the half-longitudinal indirect effect design recommended for two-wave panel data (Cole & Maxwell, 2003). Although MA was assessed at both time points (T1–T2 stability: r = 0.41, *p* < 0.01), it was anchored at T1 in the structural model, consistent with Cole and Maxwell’s (2003) recommendation that the predictor be fixed at the initial wave to preserve the prospective logic of the design. Including T2 MA as an additional predictor would risk multicollinearity with T1 MA and would reframe the model from a prospective to a contemporaneous design. For the anxiety construct, the two theoretically defined subscale scores (MLA and MEA) were used as parceled indicator variables, a widely accepted approach for multidimensional constructs to improve model parsimony and indicator reliability (Little et al., 2002). MI and SC were modeled with their individual items as indicators, given their unidimensional structure.

Model fit was evaluated using a battery of indices: the ratio of chi-square to degrees of freedom (χ^2^/δf), the Comparative Fit Index (CFI), the Tucker–Lewis Index (TLI), the Normed Fit Index (NFI), the Incremental Fit Index (IFI), the Goodness-of-Fit Index (GFI), the Root Mean Square Error of Approximation (RMSEA), and the Standardized Root Mean Square Residual (SRMR). Values of CFI, TLI, NFI, IFI, and GFI at or above 0.95 indicate good fit; values at or above 0.90 indicate acceptable fit (Hu & Bentler, 1999). RMSEA values below 0.06 reflect good fit and values below 0.08 indicate acceptable fit. The indirect effect (i.e., the indirect pathway from T1 MA through T2 MI to T2 SC) was estimated using 5000 bootstrap resamples with bias-corrected 95% confidence intervals; a confidence interval not encompassing zero was taken as evidence of a statistically significant indirect effect.

## 4. Results

### 4.1. Descriptive Statistics and Bivariate Correlations

Table 1 presents the descriptive statistics, normality indicators, reliability coefficients, and bivariate correlations for all study variables at T1 and T2. Across both time points, academic self-concept and mathematical interest were positively and significantly correlated with each other (all *p* < 0.01), whereas both constructs were significantly and negatively correlated with mathematical anxiety. These cross-sectional associations were consistent in direction and magnitude across T1 and T2, providing initial evidence of construct coherence. Stability correlations between T1 and T2 scores were substantial for all three constructs, reflecting the high autoregressive paths documented in prior longitudinal research with similar constructs (J. Zhang et al., 2023).

### 4.2. Measurement Model Evaluation

Before testing the structural model, the measurement models for each wave were evaluated separately via CFA. The Wave 1 measurement model demonstrated good fit to the data: χ^2^(17) = 40.367, χ^2^/df = 2.375, CFI = 0.988, TLI = 0.981, NFI = 0.980, IFI = 0.988, GFI = 0.981, RMSEA = 0.050, SRMR = 0.019. The Wave 2 measurement model likewise indicated good fit: χ^2^(17) = 47.093, χ^2^/df = 2.770, CFI = 0.986, TLI = 0.977, NFI = 0.979, IFI = 0.986, GFI = 0.979, RMSEA = 0.057, SRMR = 0.019. All factor loadings were statistically significant (*p* < 0.001) and of satisfactory magnitude, supporting the construct validity of each measurement model before proceeding to structural estimation.

### 4.3. Two-Wave Cross-Lagged Panel Model

**Figure 1:**
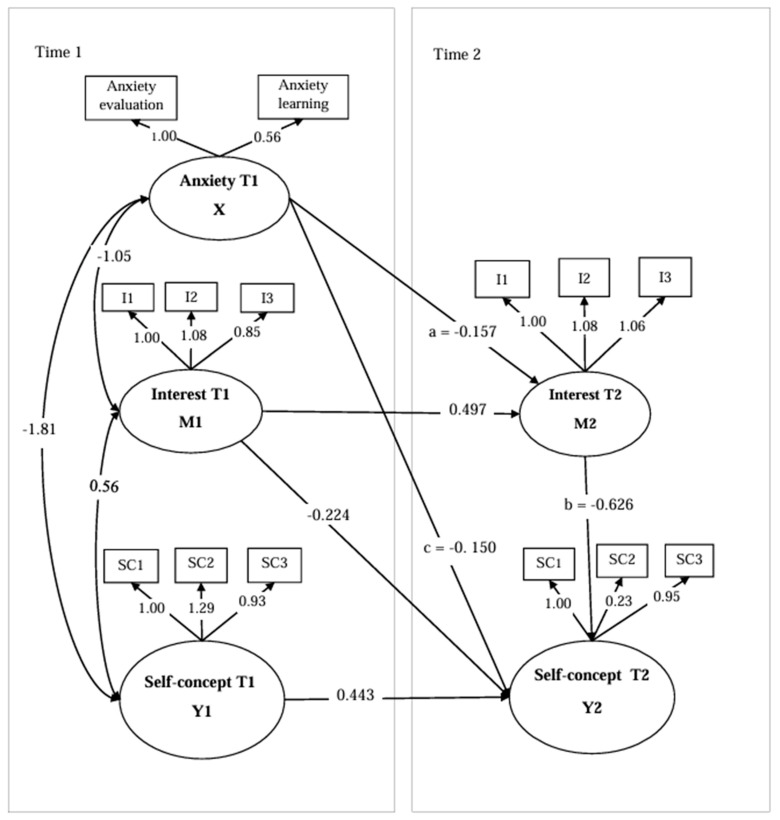
A Two-wave Cross-lagged Panel Model of MA, MI, and SC. Note. Standardized path coefficients are shown. Dashed lines represent autoregressive stability paths. Solid lines represent cross-lagged paths. *p* < 0.01.

The cross-lagged panel model examining the indirect role of MI in the MA–SC relationship was estimated with autoregressive paths included for all three constructs. The overall model fit was acceptable: χ^2^(68) = 159.030, χ^2^/df = 2.339, CFI = 0.979, TLI = 0.972, NFI = 0.964, IFI = 0.979, GFI = 0.959, RMSEA = 0.050, SRMR = 0.033. All fit indices met or exceeded the threshold for acceptable fit, and RMSEA fell within the range indicative of good fit. Figure 1 presents the standardized path coefficients of the two-wave cross-lagged panel model. Addressing H2, Path a (T1 MA → T2 mathematical interest, controlling for T1 interest) was statistically significant and negative (β = −0.157, *p* < 0.01), indicating that higher levels of MA at T1 prospectively predicted lower levels of mathematical interest at T2, above and beyond the autoregressive stability of interest itself. Addressing H3, Path b (T2 mathematical interest → T2 academic self-concept, controlling for T1 academic self-concept and T1 interest) was statistically significant and positive (β = 0.626, *p* < 0.01), demonstrating that higher levels of mathematical interest at T2 were associated with stronger academic self-concept at T2, net of all baseline levels. Addressing H1, the direct cross-lagged path from T1 MA to T2 academic self-concept (Path c; controlling for T1 academic self-concept) was also significant and negative (β = −0.150, *p* < 0.01), confirming that anxiety exerts an independent prospective influence on self-concept beyond what is mediated through interest. Addressing H4, the indirect effect of T1 MA on T2 academic self-concept via T2 mathematical interest was negative and statistically significant (β = −0.098, 95% CI [−0.167, −0.034]). Because the bootstrap confidence interval did not include zero, the hypothesized indirect effect was supported. The coexistence of a significant direct path and a significant indirect path indicates partial indirect association: mathematical interest transmits a portion of the negative influence of anxiety on self-concept, while anxiety also retains an independent direct effect on self-concept. These results fully support H1, H2, H3, and H4.

## 5. Discussion

The present study investigated the prospective indirect role of MI linking MA to SC in a sample of Turkish middle school students, using a two-wave cross-lagged panel design with a four-month measurement interval. All four hypotheses were supported. MA at T1 negatively predicted SC at T2, above and beyond baseline SC levels (H1). MA at T1 negatively predicted MI at T2, above and beyond baseline MI levels (H2). MI at T2 positively predicted SC at T2, above and beyond baseline SC and MI levels (H3). Critically, MI functioned as a partial indirect pathway in the MA–SC relationship (H4), with the indirect effect remaining statistically significant after bootstrapping. Together, these findings provide one of the first two-wave panel tests of the MA–MI–SC indirect pathway and extend prior pairwise longitudinal research by demonstrating that intrinsic motivational engagement functions as a mediating mechanism through which early anxiety shapes students’ mathematical self-perceptions over time.

The first finding, that MA at T1 negatively predicted SC at T2 after controlling for baseline SC (H1), is consistent with the substantial longitudinal literature. Ahmed et al. (2012) documented reciprocal negative relations between math SC and MA across a single academic year, and J. Zhang et al. (2023), in a three-wave study, replicated the prospective anxiety-to-self-concept pathway among East Asian adolescents. The present study extends these findings to a Turkish middle school context, indicating that the MA–SC dynamic is not restricted to high-income Western samples but also emerges in a national context characterized by elevated MA and below-average mathematics performance (Kul et al., 2024). From a CVT perspective (Pekrun, 2006), this result aligns with the prediction that anxiety, as a negative activating emotion, undermines control appraisals—students’ perceived competence and sense of mastery—thereby weakening the evaluative self-beliefs that constitute SC. The persistence of this direct effect after including MI as a mediator (β = −0.150) suggests that MA exerts an independent prospective influence on SC beyond motivational pathways. This may reflect additional mechanisms such as rumination, working memory disruption, and avoidance behaviors, which warrant further investigation. It is also noteworthy that changes in SC are likely shaped not only by anxiety but also by cumulative experiences of academic success and failure, including formal assessments. In line with the skill-development hypothesis (Marsh & Martin, 2011), prior achievement contributes to SC through social comparison processes. Future research incorporating objective achievement indicators would enable a more comprehensive decomposition of the mechanisms underlying SC development over time.

The second finding, that MA negatively predicted subsequent MI (H2; β = −0.157), is consistent with CVT’s specification that anxiety suppresses value appraisals and thereby weakens positive activity emotions, including enjoyment and interest, that sustain voluntary engagement (Pekrun, 2006; Forsblom et al., 2022). Forsblom et al. (2022) demonstrated across three waves that perceived value prospectively predicts enjoyment, whereas low perceived competence predicts boredom and anger. The present finding extends this pattern by establishing a longitudinal link between MA and MI in a middle school sample experiencing the normative motivational decline reported by Sakaki et al. (2024). Ahmed (2018) similarly showed that trajectories of high or increasing anxiety are associated with reduced interest and greater STEM disengagement during adolescence. The magnitude of the present cross-lagged effect (β = −0.157) is modest but meaningful given the high autoregressive stability of MI over a four-month interval, consistent with Gogol et al.’s (2016) observation that cross-construct longitudinal effects are typically small relative to stability paths. From an EVT perspective, these results suggest that MA undermines the intrinsic task value underlying MI, thereby initiating the motivational withdrawal process described in expectancy–value frameworks. Wang et al. (2015) further demonstrated that the effects of MA on mathematics learning are moderated by motivational engagement, suggesting that MI may play a role in shaping the strength of anxiety-related academic outcomes. This possibility warrants further longitudinal investigation.

The third finding, that MI positively predicted SC at T2 after controlling for baseline levels (H3; β = 0.626), yielded the strongest cross-lagged coefficient in the model. This large effect is theoretically coherent: within CVT, positive activity emotions and intrinsic value appraisals jointly sustain engagement, effort investment, and the accumulation of successful mathematical experiences that in turn strengthen competence beliefs. Within SDT, intrinsic motivation, of which MI is the affective manifestation, supports perceived competence through autonomous engagement, reinforcing the self-beliefs that constitute SC. Denissen et al. (2007), in a longitudinal study of elementary school children, documented simultaneous couplings among domain-specific achievement, SC, and interest, showing that these constructs reinforce one another over time. Empirically, Denner et al. (2019) showed cross-lagged effects from SC to MI across the elementary-to-middle school transition; the present finding demonstrates the reverse prospective path from MI to SC within middle school, suggesting that the relationship between the two constructs is developmentally reciprocal and that MI is not merely a downstream consequence of SC but an active upstream predictor of it. Marsh et al. (2005) documented reciprocal relations among SC, interest, and achievement across multiple adolescent waves, and the present result is consistent with that broader framework. Viljaranta et al. (2014) similarly found reciprocal dynamics between interest and SC in mathematics across Grades 1 through 7, with interest emerging as an active predictor of SC particularly in the middle school years. The strength of this path further suggests that even modest increases in MI sustained over a semester may carry substantial dividends for students’ mathematical self-perceptions.

The fourth and central finding of this study is that MI partially transmitted the prospective negative association from MA to SC (H4; β = −0.098, 95% CI [−0.167, −0.034]). Partial indirect association, indicated by the coexistence of significant direct and indirect paths, implies that MI is one of several mechanisms through which MA erodes SC, rather than the sole pathway. This partial indirect association pattern is consistent with Lei et al. (2025), who found that mathematics SC partially mediated the effect of MA on calculation fluency in Chinese elementary students, with both significant direct and indirect paths retained, indicating that anxiety operates through multiple simultaneous routes to academic outcomes. The present indirect effect, though modest in standardized magnitude, is substantively important because it identifies a modifiable motivational mechanism operating within a single academic semester: interventions that interrupt the MA-to-MI pathway early in the semester can in principle prevent a portion of the downstream erosion of SC. D. Zhang and Wang (2020) reported analogous mediation in a cross-sectional Chinese middle school sample, and Broda et al. (2023) identified the co-occurrence of high MA, low MI, and low SC as the most disadvantaged motivational profile in their CVT-based cluster analysis. The present study advances both findings by establishing that MI functions as a prospective indirect pathway in the MA–SC association, providing temporal ordering evidence that moves beyond cross-sectional co-occurrence findings.

### 5.1. Implications

The present findings carry both theoretical and practical implications. Theoretically, this study supports an integrated motivational account of the MA-SC relationship in which CVT converges on a common prediction: MA degrades value appraisals and intrinsic engagement, which in turn undermines the competence beliefs constituting SC. The observed indirect effect model provides a prospective temporal sequence consistent with Schukajlow et al.’s (2023) call for theoretically integrated accounts of emotion and motivation in mathematics, and positions MI as a proximal target for understanding how early MA initiates lasting motivational trajectories.

Practically, these results underscore the importance of intervening simultaneously on MA and MI rather than targeting either construct in isolation. Reducing MA through well-evidenced approaches such as expressive writing before assessments, reappraisal training, and low-stakes formative evaluation practices may interrupt the MA-to-MI pathway identified in this study, thereby protecting students’ intrinsic engagement. Equally, instructional strategies that enhance situational MI, including collaborative problem-solving, real-world mathematical contexts, and autonomy-supportive teaching, may buffer the erosion of SC that follows from sustained MA, even before anxiety itself is fully reduced. The finding that the MI-to-SC path was the strongest in the model (β = 0.626) suggests that fostering MI may be a particularly high-leverage point for strengthening SC in anxious learners, consistent with the implications drawn by Broda et al. (2023) and Munoz et al. (2025) from CVT-based profiling research. For school counselors and mathematics teachers in Türkiye, where PISA data document high rates of MA and below-expected engagement (OECD, 2013), these findings advocate for integrative classroom-level interventions that simultaneously address affective safety and intrinsic motivation as co-determinants of students’ mathematical self-perceptions. Peixoto et al. (2017) further demonstrated that competence and value appraisals jointly predict achievement emotions and academic outcomes in mathematics, providing additional empirical support for the integrated CVT–EVT framework adopted here.

### 5.2. Limitations and Future Research

Several limitations of the present study should be acknowledged. First, the two-wave design, while sufficient to establish temporal ordering of effects and offering stronger temporal inference than cross-sectional designs, does not permit examination of the developmental dynamics of the MA–MI–SC system across multiple transitions. All three constructs exhibit high autoregressive stability (Gogol et al., 2016; J. Zhang et al., 2023), and the residual cross-lagged variance captured within a single four-month interval may not fully reflect the cumulative developmental mechanisms operating across the middle school years. Future research should employ three- or four-wave designs spanning the full Grades 6–8 period to examine whether the indirect associative pathway strengthens, weakens, or reverses direction as students progress through the normative motivational decline documented in this age range (Sakaki et al., 2024). Furthermore, the present study employed a standard cross-lagged panel model, which cannot fully disentangle stable between-person differences from within-person change over time (Hamaker et al., 2015). Recent work by Paschke et al. (2025) in a mathematics motivation context demonstrated that random-intercept cross-lagged panel models (RI-CLPMs) may yield more conservative cross-lagged effect estimates relative to standard models. Future research should therefore examine whether the MA–MI–SC indirect pathway replicates under this more conservative analytical framework. Additionally, although MA was measured at both time points, the omission of T2 anxiety from the structural model is acknowledged as a limitation; three-wave designs would permit MA to be modeled as a dynamic process rather than a static baseline predictor.

Second, all measures relied on student self-report, which introduces the possibility of shared method variance inflating observed correlations and social desirability bias attenuating MA reports. Future research should complement self-report data with behavioral indicators of mathematical engagement, such as voluntary task persistence, help-seeking frequency, and elective mathematics participation, as well as teacher or observer ratings, in order to triangulate the MI construct. The sample was drawn from a single province in Turkey and consisted exclusively of middle school students, limiting generalizability to other Turkish regions, educational levels, and cultural contexts. Replication with samples from different geographic regions, school types, and socioeconomic backgrounds would strengthen the external validity of the proposed mediation model. Additionally, because the present study did not collect gender or socioeconomic information, the potential moderating roles of these variables on the MA–MI–SC pathways could not be examined. Given evidence that gender moderates aspects of the MA–SC relationship in some populations (Wang et al., 2020), future studies should investigate whether the mediation model operates equivalently across demographic subgroups.

Third, the present study examined a single mediating variable. Given that CVT proposes multiple emotional and motivational pathways connecting MA to academic outcomes (Pekrun, 2006, 2024), future research may benefit from investigating whether mathematics self-efficacy, achievement emotions other than anxiety (e.g., boredom, enjoyment), or broader indicators of academic engagement function as additional or competing mediators within the same longitudinal framework. A serial mediation model incorporating both MI and self-efficacy as sequential mediators between MA and SC would permit a more comprehensive test of the motivational cascade implied by this theoretical framework.

Fourth, beyond model complexity, the present study assessed SC as a relatively stable construct measured at two distal time points. However, prior research using experience-sampling methodology has demonstrated that mathematics self-concept may fluctuate meaningfully across instructional contexts in response to perceived achievement (Niepel et al., 2022). Future research employing intensive longitudinal or experience-sampling designs could therefore capture more fine-grained within-person dynamics in the MA–MI–SC system and determine whether the indirect associative pathway identified here operates similarly across shorter timescales. Another limitation concerns the absence of mathematics achievement as an outcome variable. Examining whether the MA–MI–SC pathway ultimately predicts mathematics grades or standardized test performance would extend the practical significance of the model and strengthen its connection to achievement outcomes central to mathematics education research.

Finally, the use of a brief MI scale warrants consideration. Although the items were adapted from the validated OECD (2003), framework and demonstrated strong internal consistency in the present sample, the relatively small number of items may limit the breadth of construct representation among early adolescents. Future studies may benefit from examining whether developmentally calibrated MI measures, incorporating expanded item sets and age-appropriate wording, yield comparable or differentiated patterns of association with MA and SC. Particular attention should be given to ensuring that item wording remains sensitive to age-related cognitive and motivational characteristics.

## 6. Conclusions

This study provides one of the early longitudinal examinations of MI as an indirect pathway linking MA to SC using a two-wave cross-lagged panel design with Turkish middle school students. The findings indicated that MA prospectively predicted declines in both MI and SC over a four-month period, while MI positively predicted subsequent SC beyond baseline levels. Moreover, MI partially mediated the negative effect of MA on SC, supporting the presence of a prospective indirect pathway operating across the academic semester. These findings are consistent with theoretical expectations derived from CVT, EVT, and SDT, and extend prior longitudinal research by demonstrating the temporal sequencing of anxiety, interest, and self-perception within a unified motivational framework.

The observed pattern of indirect effects further suggests that MA influences students’ mathematical self-perceptions through both motivational mechanisms, reflected in reductions in MI, and additional pathways that may involve cognitive and affective processes not directly captured in the present model. From an applied perspective, these findings indicate that interventions targeting MA should not be limited to anxiety reduction alone but should also incorporate strategies that foster students’ intrinsic interest in mathematics. Supporting MI may function as a protective factor for maintaining positive SC, particularly in educational contexts where sustained anxiety poses a risk to students’ long-term engagement with mathematics. More broadly, the present findings underscore the importance of integrated intervention frameworks that simultaneously address emotional regulation, motivational development, and self-perception in mathematics learning environments.

## Figures and Tables

**Table 1:** Descriptive Statistics, Normality Indicators, Reliability Coefficients, and Correlations Among Study Variables.

Variable	1	2	3	4	5	6
1. Self-Concept T1	*–*					
2. Self-Concept T2	0.59 **	–				
3. Interest T1	0.58 **	0.39 **	–			
4. Interest T2	0.40 **	0.64 **	0.45 **	–		
5. Anxiety T1	−0.42 **	−0.39 **	−0.25 **	−0.23 **	–	
6. Anxiety T2	−0.29 **	−0.40 **	−0.10 *	−0.30 **	0.41 **	–
Mean	10.00	10.10	10.45	9.81	22.18	21.38
SD	2.98	3.02	3.05	2.97	7.33	6.75
Skewness	−0.205	−0.386	−0.462	−0.416	0.363	0.402
Kurtosis	−0.477	−0.248	−0.456	0.019	−0.169	0.506
McDonald ω	0.90	0.90	0.81	0.83	0.86	0.87
Cronbach α	0.89	0.90	0.81	0.83	0.81	0.82
Guttman λ6	0.85	0.86	0.74	0.77	0.83	0.84

* *p* < 0.05, ** *p* < 0.01.

## Data Availability

The data presented in this study are openly available in Figshare at https://doi.org/10.6084/m9.figshare.31869988.
